# Assessment of suicidal ideation via telemedicine: a case report and management suggestions

**DOI:** 10.1186/s12245-023-00557-2

**Published:** 2023-11-13

**Authors:** Tarso Augusto Duenhas Accorsi, Karine De Amicis Lima, Karen Francine Köhler, Eduardo Cordioli, Carlos Henrique Sartorato Pedrotti

**Affiliations:** Hospital Israelita Albert Einstein, Secretaria da Unidade de Telemedicina, Av. Albert Einstein, 627. Bloco B, 2° Andar, Sao Paulo, 05652-900 Brazil

**Keywords:** Telemedicine, Suicide risk, Risk management, Patient safety, COVID-19 pandemic

## Abstract

**Background:**

Nowadays, we find ourselves in very unexpected and challenging circumstances facing the COVID-19 pandemic. The impact of the new coronavirus pandemic probably affected everyone’s mental health, and people with pre-existing mental disorders may have an aggravated disease condition, leading to a suicide attempt. Pandemic also increased the use of direct-to-consumer telemedicine (TM) exponentially, and consequently, it was expected that cases of attempted suicide could be evaluated remotely. Some TM centers have adapted safety protocols from psychiatric guidelines for managing these patients. However, there is a lack of evidence of the effectiveness of follow-up by TM for patients at high risk for suicide, and there is no consensus on what action should be taken vis-à-vis the patient who requests immediate help remotely.

**Case presentation:**

Here, we reported a case of a TM evaluation of a patient’s suicidal ideation in a direct-to-consumer telemedicine emergency center, describing the conduct taken in the face of this situation. We also discuss the importance of planning the emergency telemedicine center for situations of risk of suicide.

**Conclusions:**

Telemedicine centers should be prepared for direct consumer assessment of suicidal ideation. Current management suggestions include recognizing the risk profile through institutional training and software skills and immediate referral for face-to-face assessment, encouraging continuous monitoring until the admission and active recruitment of family members or closest friends.

## Background

Suicides account for 1.4% of premature deaths worldwide [1]. Suicidal ideation (SI) increases the risk of complete suicide from 2.35 to 8 times, especially in psychiatric patients [2, 3]. COVID-19 pandemic is multifactorial related to increased suicide rates [4].

Pandemic also increased the use of direct-to-consumer telemedicine (TM) exponentially, and consequently, it was expected that cases of SI could be evaluated remotely [5]. Among several suicide behavior prevention strategies, the effect of providing a helpline support, including telemedicine, is still unclear. TM assessment may have some advantages compared to face-to-face consultation; for example, it requires less patient commitment, is less stigmatizing, and is more suitable for psychomotor impairment. However, some inconveniences may exist, like less nonverbal manifestation and more artificial relationships [6].

Some TM centers have adapted safety protocols from psychiatric guidelines for managing these patients [7, 8]. But there is a lack of evidence of the effectiveness of follow-up by TM for patients at high risk for suicide, and there is no consensus on what action should be taken vis-à-vis the patient who requests immediate help remotely [9].

Despite psychiatric TM center being associated with the rapid referral of patients at risk of suicide in remote areas, in a general direct-to-consumer TM emergency center, healthcare providers are unaccustomed and untrained to virtual psychiatric care and caring for a patient SI can be challenging [10].

This pandemic provoked unprecedented public health actions to prevent the spread of the virus: confinement of more than half of the world’s population, closure of schools and universities, social and physical distancing, and the declaration of health emergencies in many countries [3, 4, 11]. Previous studies have shown that infectious disease outbreaks are associated with mental health symptoms and disorders (e.g., depression, anxiety, posttraumatic stress disorder (PTSD), insomnia) in survivors, family members, healthcare workers, and members of affected communities [12–16]. A meta-analysis has shown the significant consequences of Ebola disease on mental health [12]. Although having a lower fatality case rate than Ebola, this pandemic is associated with many deaths worldwide. Studies conducted among affected populations have shown significant risk factors for the mental health of affected people [17]. Compared to what was observed in the latest WHO study on common mental health disorders, the prevalence of depression in populations affected by COVID-19 is more than three times higher (15.97%) than in the general population (4.4%), while it is four times higher for anxiety (15.15% vs. 3.6%) and five times higher for PTSD (21.94% vs. 4%) [18–21].

The brutal and lasting turnaround in rhythms, habits, and social relationships require unprecedented adaptability on both an individual and collective scale, and the healthcare system must adapt to promote the best possible continuity of care. Regular communication is mainly affected by confinement and detachment. Mental health professionals must ensure that this communication is maintained through the appropriate means [17]. Global containment in the context of the COVID-19 pandemic forces the mental health system into new modes of operation to reach the general population without habitual access to care. These adaptive capacities will have to be continued in the long term because the exit from confinement will bring new challenges with updating disorders that have remained distant from care or the appearance of “posttraumatic” disorders [22, 23].

This article is a case report of a TM evaluation of a patient’s SI in a direct-to-consumer emergency center. The remote general practitioner attended a female patient with a history of depression without proper treatment and clearly identified a high risk of suicide. The medical conduct taken in this situation is set out in detail. The patient was referred immediately and remained accompanied remotely until face-to-face evaluation.

This report aims to demonstrate the importance of planning the emergency telemedicine center for situations of risk of suicide, which involves the training of professionals, provision of adapted online guidelines, use of software advantages, a set of checklists of necessary procedures, and the possibility of readjusting the service after a long-run displacement of a professional for this situation. In addition, there is a demonstration of TM staff’s importance of well-trained technical and behavioral skills and TM’s reinforcement as an effective way to first assess patients in the healthcare system.

## Case presentation

A female patient, 23 years old, located in the same city as the medical center (São Paulo, SP, Brazil), requested TM evaluation on February 07, 2021, at night. A senior general practitioner from this TM center answered the call promptly. The patient requested a connection from their LG K10 cell phone (Android, 5.3-in. screen) using specific software developed by TM Center called Einstein Conecta, version 1.0. The remote doctor was working from home using a personal computer, accessing the same software.

The symptom complaint and its duration were vague: malaise today. Spontaneously, the patient reported feeling unwell, weakness, and mild headache. When actively asked, no localizing symptoms or acute disease hypotheses were presented. These symptoms have been recurring spatially since the beginning of the COVID-19 pandemic. There was no fever, chills, myalgia, arthralgia, upper airway symptoms, cough, dyspnea, chest pain, abdominal pain, nausea, or urinary abnormalities. The patient’s face was in an altered mood and apparent recent crying. The remaining remote physical examination showed the good subjective general condition, regular breathing pattern at rest, normal speech, and behavior, with no signs of mental confusion, no skin or mucous discoloration, no jaundice, no cyanosis, no signs of dehydration or tissue hypoperfusion, and no signs of intense pain. When asked for personal history, initially, no antecedent was highlighted, but the question about current medicine was positive: she was on sertraline 50 mg once a day since a few months ago. She was not pregnant and had no allergies. She does not have a regular medical follow-up.

After a complete remote questionnaire, the doctor checked previous TM encounters and, surprisingly, found seven recent evaluations. The first of these assessments occurred 5 months ago, followed by three reviews approximately 1 month apart and the last three visits in the previous month. The final ICD-10 diagnosis was utterly different among them, and, except for headache, they were not noticed in the present evaluation: muscle pain, anxiety, asthma, limb pain, and articular pain. Moreover, all encounters were at night.

Based on this compilation of information, the doctor suspected an unadjusted psychiatric condition. A deep depression anamnesis was started, and a lot of typical symptoms were manifested in the last few weeks: anhedonia, sadness, feeling of worthlessness, feeling of ruin, lack of energy, and reported somatic symptoms. The next step was a direct question, but it was welcomed if the patient thought about SI. She immediately replied, “yes!”. After this point, she felt free to talk about her condition, and despite much crying, she felt welcomed and understood. She confirmed recurrent suicidal thinking, a sense of self-harm but never attempted it objectively. On this day, the symptom was the most intense she ever had. The doctor slowly explained the importance of asking for expert help and the possibility of an excellent prognosis after the correct treatment. This information encouraged the patient, who positively altered her facial expression and was motivated to follow the recommendation.

A safety point was the interrogation about family members or other companies at that moment at home. She was alone, unemployed, and 2 km far from the next relative, a cousin. It was another red flag in this remote evaluation. The only strategy to provide this patient immediate and correct assessment would be to refer for face-to-face assessment at the emergency department. There could be a clinical and toxic-metabolic and a psychiatric inter-consultation evaluation.

Faced with the difficulties found, the doctor insisted that the patient access the closest family member and made the company available via TM throughout the journey to the emergency department and even with a duty physician’s report. The objective was successful, and the patient was admitted for a complete evaluation. Unfortunately, there is no more data from this evaluation point.

## Discussion

Suicide is the tenth leading cause of death in developed countries, with around 800,000 cases per year worldwide, and for each case, there are generally two SI [24]. Selective and universal interventions are required for suicide prevention. TM can screen factors such as financial stress, domestic violence, alcohol consumption, isolation, and access to means of act [6].

In the USA, 650,000 patients directly seek emergency care for suicidal behavior annually [25]. With the progressive incorporation of TM into the health system, including emergency settings, a progressive increase in the remote evaluation of patients with suicidal ideation is expected. Both emergency assessment strategies (face-to-face or virtual) should have two objectives: identifying patients at higher risk for suicide and applying strategies to reduce this risk [26]. However, different from other life-threatening conditions, emergency providers did not have sufficient training in mental health issues, and they usually tend to rely extensively on psychiatric consultant recommendations [27]. Beyond this, the screening of at-risk suicide patients is too complex among thousands of non-psychiatric complaints, mainly in remote evaluation. Paradoxically, TM evaluation may offer advantages regarding this issue, as the possibility of supporting artificial intelligence assistance and simple software support assistance, on-time guidelines consultation during the conversation with the patient, and the condition of the physician’s environment that provides better care to patients with psychiatric complaints [28]. A Natural Language Processing (NLP) Algorithm was able to detect suicidal content in patients’ written communication to their therapists, which can potentially be replicated in any context [29]. Even when a patient seeks an in-person assessment, digital health strategies are encouraged to build connections, potentially improving the patient’s openness to providers [30].

In this case report, only after seven TM encounters the SI was recognized. It is a fact that implies the need for a TM center leads up in this issue. After this episode, there was feedback to the institution, and several improvement strategies were discussed.

Firstly, the number of SI in this TM center was screened, detecting that our center experienced a boost in suicide ideation evaluation during the pandemic: from 30 cases in 2020 to 6 cases per month in 2021. This data highlights the issue’s importance. Some evidence has confirmed that using appropriate recommendations and guidelines, on-demand telepsychiatry has been proven to provide continuity of mental health care and reduce emergency department pressure, discharge times, and psychiatric hospitalization rates. Depending on the expansion and capacity of the TM Centers, the provision of remote psychiatric assessment may be made available [31].

Secondly, suicide risk factors from medical literature were compiled. At-risk patients’ identification begins with recognizing previous SI and mental health (especially depression) or substance use (alcohol or drugs) disorder [32]. Population risk profile is variable in each country, but there is a universal tendency to be more prevalent in racial and sexual behavior in minorities, in people with physical disabilities, chronic pain, and some particular occupations (agricultural, physicians, and attorneys) [33]. Assessment of the level of suicide risk is challenging, but it is the turning point in indicating inpatient or outpatient treatment. In the absence of promptly available mental health consultants, in addition to mental history, a key issue is the identification of protective factors, essentially close companions and easy access to the health system [34]. For suicidal crisis trigger recognition, it is important to emphasize the role of anamnesis in assessing the patient’s current life context and not only the distal suicidal risk factors.

The institutional TM training on these clinical aspects is a cornerstone. Encouraging the SI approach and deepening the anamnesis is fundamental. Asking about SI does not increase the risk of suicide and allows the patient to ask for help [27]. Red flag signs compiled by artificial intelligence may help create an alert box for the physician. More simply, provided adapted guidelines can be online checked, and the electronic medical record may have checking boxes and signaling tools to support assistance. Notably, the patient had several risk factors in this case, but there was no previous deep anamnesis. One single positive risk factor may imply using a checklist of questions for suicide ideation. We understand that a TM evaluation must be very sensitive in this issue. Any suicide risk factor must lead to face-to-face evaluation. Even in alternative patient conduct, it must be observed in the emergency department or psychiatric professional bedside.

Moreover, there was a discussion of management strategies. There is no evidence of the safety of complete remote management of a suicidal ideation patient, and the focus of the TM assessment is recognition, reception, and follow-up until immediate face-to-face evaluation [35]. It is essential to highlight that although a SI raises the risk of future suicide death, the correct referral and treatment reduce the chance of death by suicide after the first attempt to less than 10% [36]. Therefore, properly managing these patients changes the prognosis, and increasingly, the initial contact of the risk patient will be virtual. Unfortunately, one of the limitations of the present study was the absence of a telepsychiatrist to refer the patient immediately.

## Conclusion

In conclusion, it is crucial to guarantee an immediate face-to-face assessment after TM SI recognition. In this kind of direct-to-consumer TM assessment based on general practitioners, we believe that—at this time—there is no safe graduated orientation. Like the exposed case report, the ideal is to persist as virtual support providing continuous monitoring of the patient’s travel to the emergency department. Active call-in case of connection drop and request for a family member or close companion should be encouraged.

Direct-to-consumer telemedicine evaluation of SI is progressively frequent. Therefore, telemedicine centers must lead up to this issue. Current management suggestions include risk profile recognition by institutional training and software skills and immediate referral for face-to-face evaluation, encouraging continuous monitoring until admission and active recruitment of family members or other closest friends.

## Data Availability

Not applicable.

## Abbreviations

COVID-19: Coronavirus disease 19
ICD: International Classification of Diseases
PTSD: Post-traumatic stress disorder
SI: Suicide ideation
TM: Telemedicine
USA: United States of America
WHO: World Health Organization
